# Association of gastroesophageal reflux disease with the incidence of pulmonary disease

**DOI:** 10.3389/fcell.2025.1552126

**Published:** 2025-07-23

**Authors:** Xin Wang, Yan Wang, Yijie Bu, Yu Liu, Sheng Gong, Guowei Che

**Affiliations:** ^1^ Department of Respiratory and Critical Care Medicine, State Key Laboratory of Respiratory Health and Multimorbidity, Key Laboratory of Plateau Medicine of Sichuan Province, Center for High Altitude Medicine, West China Hospital, Sichuan University, Chengdu, China; ^2^Department of Thoracic Surgery/Lung Cancer Center, West China Hospital, Sichuan University, Chengdu, China; ^3^Department of Thoracic Surgery, The Public Health Clinical Center of Chengdu, Chengdu, China

**Keywords:** gastroesophageal reflux disease, pulmonary disease, meta-analysis, asthma, pneumonia

## Abstract

**Objective:**

Concurrent pulmonary diseases are common in patients with gastroesophageal reflux disease (GERD). However, whether GERD increase the incidence of pulmonary diseases is uncertain because of a lack of quantitative evidence. We conducted a meta-analysis to determine whether GERD was associated with the increased incidence of subsequent of pulmonary diseases.

**Methods:**

The PubMed, Embase, Web of Science and Cochrane Library databases were searched through 12 July 2024. The primary outcomes were asthma and pneumonia, and the secondary outcomes were pulmonary fibrosis (PF), chronic obstructive pulmonary disease (COPD), lung cancer, interstitial lung disease (ILD), bronchiectasis, bronchitis, acute lung injury (ALI), pulmonary embolism, pulmonary tuberculosis (PTB) and nontuberculous mycobacterial pulmonary disease (NTMPD). Odds ratios (ORs) with 95% confidence intervals (CIs) were calculated to investigate the associations of prior GERD with the incidence of pulmonary diseases, and subgroup analyses based on the treatment of GERD, age and source of OR were performed.

**Results:**

A total of 45 cohort studies were included. The pooled results indicated that GERD was significantly linked to an increased incidence of asthma (OR = 1.50, P < 0.001) and pneumonia (OR = 1.53, P < 0.001), as did PF (OR = 1.43, P = 0.001), COPD (OR = 1.41, P = 0.004), lung cancer (OR = 1.51, P < 0.001), ILD (OR = 1.28, P = 0.015), bronchiectasis (OR = 1.63, P = 0.039), bronchitis (OR = 1.24, P < 0.001), ALI (OR = 2.07, P < 0.001), pulmonary embolism (OR = 1.33, P = 0.013), PTB (OR = 1.63, P = 0.015) and NTMPD (OR = 3.36, P < 0.001). Subgroup analyses stratified by age and source of OR yielded similar results. However, no significant associations between treated GERD and the incidence of asthma (OR = 1.27, P = 0.081) or lung cancer (OR = 1.01, P = 0.97) were observed.

**Conclusion:**

The presence of GERD is associated with an increased incidence of subsequent various pulmonary diseases, but regular treatment may eliminate this effect. These findings highlight the importance of screening and management for pulmonary diseases and of standardized therapy in patients with GERD.

**Clinical trial registration no:**

INPLASY202490013

## Introduction

Gastroesophageal reflux disease (GERD) is a common digestive disorder characterized by the reflux of stomach contents into the esophagus, leading to a variety of symptoms and complications (Azer et al., 2024). Globally, the prevalence of GERD varies by region and diagnostic criteria, but GERD is generally estimated to affect approximately 10%–20% of adults (Richter and Rubenstein, 2018; Rubenstein and Chen, 2014). The prevalence of GERD is relatively high in Western countries but relatively low in other regions, such as Asia and Africa (Rubenstein and Chen, 2014; Jung, 2011). However, in recent years, the prevalence of GERD in China has been increasing due to changes in lifestyle and dietary habits (Liu et al., 2023; Li et al., 2023a; Zhang et al., 2022). The risk factors for GERD include advanced age, obesity, smoking, alcohol consumption, and a high-fat diet (Richter and Rubenstein, 2018). GERD can significantly impact quality of life and increase the risk of complications such as esophagitis, Barrett’s esophagus, and esophageal adenocarcinoma (Azer et al., 2024).

GERD is often overlooked and misdiagnosed in clinical practice, primarily because of atypical symptoms and a lack of awareness about the need for individuals with these symptoms to seek medical consultation and treatment. The standardized treatment of GERD requires long-term medication and improvements in lifestyles. However, a significant number of patients are unable to comply, leading to the development of long-term complications (Katzka, 2014). The impact of GERD on the risk of developing the abovementioned esophageal disorders has been well documented (Azer et al., 2024; Abdallah et al., 2019). In recent years, accumulating evidence has suggested that GERD may also have a significant effect on the development of lung diseases such as the asthma and pulmonary fibrosis which severely affect the quality of life and increase the mortality among GERD patients (Azer et al., 2024). However, this issue has not yet been thoroughly explored or systematically addressed. Clarifying the impact of GERD on pulmonary diseases can further reveal the underlying mechanisms of these diseases, while also highlighting the role of treating GERD in the prevention and management of respiratory diseases.

Therefore, this study aimed to clarify the impact of GERD on the incidence of pulmonary diseases on the basis of available evidence. The findings will contribute to the clinical management of patients with GERD, as well as to the prevention and early screening of pulmonary diseases.

## Materials and methods

This meta-analysis was registered with the International Platform of Registered Systematic Review and Meta-analysis Protocols (INPLASY202490013, DOI: 10.37766/inplasy2024.9.0013).

### Literature search

PubMed, Embase, Web of Science and Cochrane Library databases were searched from database inception through 12 July 2024 for the following terms: gastroesophageal reflux, gastro-oesophageal reflux, chronic obstructive pulmonary disease, COPD, asthma, pulmonary tuberculosis, pneumonia, pulmonary fibrosis, pulmonary embolism, lung cancer, bronchitis, bronchiectasis, pulmonary disease, respiratory disease, risk, incidence and morbidity. The search strategy is presented in Supplementary file 1. During the search, MeSH terms and free texts were applied, and references in the included studies were also reviewed.

### Inclusion criteria

Studies that met the following criteria were included: 1) GERD was diagnosed on the basis of symptoms such as acid reflux and heartburn, as well as through analysis by gastroscopy, PPI test or 24-h esophageal pH monitoring both among the adults and children; 2) GERD was diagnosed before the occurrence of pulmonary diseases, but in cases where the original articles did not explicitly report whether GERD preceded pulmonary disease, we inferred the sequence based on the study’s hypothesis or statements such as “GERD increases the risk of” or “GERD is a risk factor for pulmonary disease”; 3) pulmonary diseases were diagnosed on the basis of symptoms, blood tests, etiology, imaging, and pathological examinations and/or bronchoscopy; 4) the incidence rates of pulmonary diseases were compared between the GERD and non-GERD groups, and represented as odds ratios (ORs) with 95% confidence intervals (CIs) or enough data were provided to calculate them; 5) full text was available; and 6) if the data severely overlapped or were duplicated, only the latest or most comprehensive studies were included.

### Exclusion criteria

Studies that met the following criteria were excluded: 1) the presence of other confounding factors, such as gastrointestinal disorders; 2) ORs with 95% CIs were not available even after the authors were contacted; 3) the endpoints were not of interest, such as exacerbations of pulmonary diseases; and 4) letter, meeting abstract, editorial, animal trial, review or case report articles.

### Literature selection and data collection

First, duplicated publications were removed automatically or manually by EndNote (version 21.3, Clarivate Analytics, London, England, United Kingdom) software. The titles and abstracts were subsequently screened for relevance, and the full texts of potentially relevant publications were further reviewed for suitability.

The following data were extracted from each included study: name of first author, publication year, data source, age, sample size, number of GERD cases, treatment history of GERD (treated vs untreated), source of OR (multivariate vs univariate), endpoint, and OR and 95% CI for the corresponding endpoint.

In this meta-analysis, based on the strength of the association between GERD and various pulmonary diseases, as well as the extent of their reporting in previous studies, the primary outcomes were defined as asthma and pneumonia, and the secondary outcomes were pulmonary fibrosis (PF), chronic obstructive pulmonary disease (COPD), lung cancer, interstitial lung disease (ILD), bronchiectasis, bronchitis, acute lung injury (ALI), pulmonary embolism, pulmonary tuberculosis (PTB) and nontuberculous mycobacterial pulmonary disease (NTMPD).

### Methodological quality assessment

All the studies included in our meta-analysis were cohort studies. Therefore, the Newcastle–Ottawa scale (NOS) was used for quality evaluation, including cohort selection, comparability and outcome measurement. Studies with an NOS score >5 were regarded as high-quality studies (Stang, 2010).

The literature search, selection, data extraction, and quality evaluation were independently performed by two authors (Yao Wang and Yijie Bu), and all disagreements and discrepancies were resolved through team discussion.

### Statistical analysis

All statistical analyses were conducted by STATA (version 15.0, StataCorp LLC, College Station, Texas, United States) software. The heterogeneity among included studies was assessed by the I^2^ statistic and Q test. When significant heterogeneity was observed, presented as I^2^>50% and/or P < 0.1, the random-effects model was used; or the fixed-effects model was applied (Barili et al., 2018). The ORs with 95% CIs were combined to evaluate the association between presence of GERD and incidence of pulmonary diseases. If ORs and 95% CIs were both reported in the multivariate and univariate analyses, data from multivariate analysis were extracted and applied preferentially. The sensitivity analysis was conducted to clarify the source of heterogeneity and evaluate the stability of the pooled results. Besides, Begg’s funnel plot and Egger’s test were performed to identify publication bias for the asthma and pneumonia (Begg and Mazumdar, 1994; Egger et al., 1997). Significant publication bias was defined as the noticeably asymmetric Begg’s funnel plot and P value < 0.05 of Egger’s test, and then the trim-and-fill method was applied to evaluate the impact of potentially unpublished studies on the stability of overall pooled results, with an inspection level of α = 0.05 (Wang et al., 2021).

Furthermore, subgroup analyses stratified by the treatment of GERD (treated vs. untreated), age (adult vs. child) and source of OR (multivariate vs. univariate) were also conducted to identify the impact of these factors on the association of GERD with incidence of pulmonary diseases.

## Results

### Literature search and selection

**FIGURE 1:**
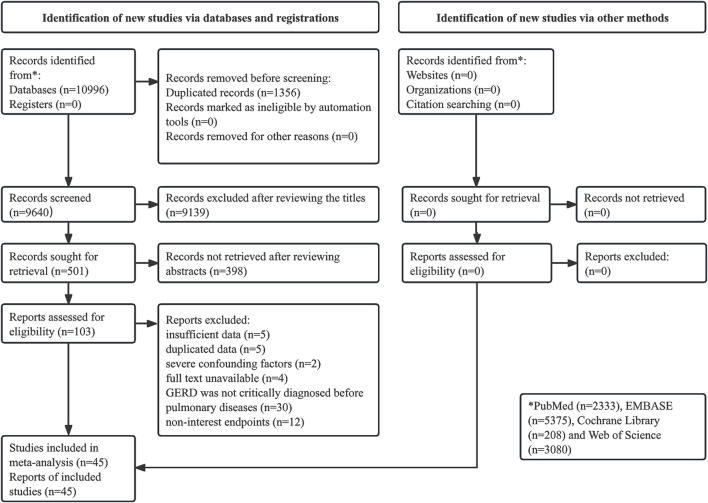
Prisma flow diagram of this meta-analysis.

The literature search and selection process were presented in Figure 1. Initially, 10,996 records were identified from the databases, and 1,356 duplicated records were removed. A total of 9,139 and 398 publications were excluded after reviewing the titles and abstracts, respectively. After the full texts were carefully reviewed, 57 publications were excluded, thirty of which were due to GERD not being critically diagnosed before the occurrence of pulmonary disease. Eventually, 45 studies were included in the meta-analysis (el-Serag and Sonnenberg, 1997; El-Serag et al., 2001; Gislason et al., 2002; Jaspersen et al., 2003; Gunnbjömsdóttir et al., 2004; Ruigómez et al., 2005; Garcia Rodriguez et al., 2008; Gribbin et al., 2009; Uddenfeldt et al., 2010; García-Sancho et al., 2011; Emilsson et al., 2013; Patria et al., 2013; Martinez et al., 2014; Wang et al., 2014; Zhong et al., 2015; Fan et al., 2016; Hsu et al., 2016; Yang et al., 2016; Hsu et al., 2017; Zhang et al., 2017; Choi et al., 2019; Kim et al., 2020a; Kim et al., 2020b; Kuo et al., 2020; Wang et al., 2020; Abdel Baseer and Sakhr, 2021; Baqir et al., 2021; Cantarutti et al., 2021; Amarnath et al., 2022; Ahn et al., 2023; Cotton et al., 2023; Duncan et al., 2023; Jareebi et al., 2023; Kim et al., 2023; Li et al., 2023b; Reynolds et al., 2023; Sarwar Zubairi et al., 2023; Shen et al., 2023; Cleven et al., 2024; Dong et al., 2024; Fakhraei et al., 2024; Liu et al., 2024; Shan and Ge, 2024.

### Basic characteristics of the included studies

These 45 cohort studies were published from 1997-2024, with sample sizes ranging from 43-602,604. Most of the participants were from public databases and were adult patients. The associations of GERD with the incidence of asthma (el-Serag and Sonnenberg, 1997; El-Serag et al., 2001; Gislason et al., 2002; Jaspersen et al., 2003; Gunnbjömsdóttir et al., 2004; Ruigómez et al., 2005; Uddenfeldt et al., 2010; Emilsson et al., 2013; Martinez et al., 2014; Kim et al., 2020a; Kim et al., 2020b; Wang et al., 2020; Cantarutti et al., 2021; Ahn et al., 2023; Fakhraei et al., 2024; Yao et al., 2024), pneumonia (el-Serag and Sonnenberg, 1997; El-Serag et al., 2001; Patria et al., 2013; Martinez et al., 2014; Wang et al., 2014; Yang et al., 2016; Hsu et al., 2017; Zhang et al., 2017; Kuo et al., 2020; Abdel Baseer and Sakhr, 2021; Dong et al., 2024), PF (el-Serag and Sonnenberg, 1997; Gribbin et al., 2009; García-Sancho et al., 2011; Baqir et al., 2021; Cotton et al., 2023; Reynolds et al., 2023; Sarwar Zubairi et al., 2023; Wu et al., 2024), COPD (el-Serag and Sonnenberg, 1997; Garcia Rodriguez et al., 2008; Shen et al., 2023; Liu et al., 2024), lung cancer (Hsu et al., 2016; Choi et al., 2019; Amarnath et al., 2022; Li et al., 2023b), ILD (Jareebi et al., 2023; Cleven et al., 2024; Shan and Ge, 2024), bronchiectasis (el-Serag and Sonnenberg, 1997; El-Serag et al., 2001; Duncan et al., 2023) and bronchitis (el-Serag and Sonnenberg, 1997; Dong et al., 2024) were explored in 16, 11, 8, 4, 4, 3, 3 and 2 of the included studies, and 1 study explored the relationships between GERD and the incidence of ALI (Zhong et al., 2015), pulmonary embolism (Dong et al., 2024), PTB (Fan et al., 2016) and NTMPD (Kim et al., 2023). The data are shown in Table 1.

**TABLE 1:** Basic characteristics of included studies.

Author	Year	Country	Data source	Age (year-old)	Sample size	Number of GERD cases	Treatment of GERD	Source of OR	Endpoints
el-Serag and Sonnenberg (1997)	1997	United States	Department of Veterans Affairs	60 ± 13/56 ± 15	202,732	101,366	NR	M	Asthma, bronchiectasis, bronchitis, COPD, PF, pneumonia
El-Serag et al. (2001)	2001	United States	Texas Children’s Hospital	9.16 ± 4.61/8.64 ± 4.92	9,900	1980	NR	M	Asthma, bronchiectasis, pneumonia
Gislason et al. (2002)	2002	Iceland, Belgium, and Sweden	Iceland, Belgium, and Sweden	33 ± 7/34 ± 7	2,197	101	NR	M	Asthma
Jaspersen et al. (2003)	2003	Germany, Austria and Switzerland	Germany, Austria and Switzerland	53.8 ± 14.0	4,179	2,114	NR	M	Asthma
Gunnbjömsdóttir et al. (2004)	2004	Iceland, Norway, Denmark, Sweden and Estonia	Iceland, Norway, Denmark, Sweden and Estonia	39.6 ± 7.1	14,552	1,422	NR	M	Asthma
Ruigómez et al. (2005)	2005	United Kingdom	United Kingdom General Practice Research Database	2–79	13,758	5,653	NR	M	Asthma
Garcia Rodriguez et al. (2008)	2008	United Kingdom	United Kingdom General Practice Research Database	40–79	9,509	4,391	NR	M	COPD
Gribbin et al. (2009)	2009	United Kingdom	The Health Improvement Network primary care database	>40	4,513	387	NR	U	Idiopathic PF
Uddenfeldt et al. (2010)	2010	Sweden	Gastrikland and Jamtland in the central part of Sweden	16–69	8,150	NR	NR	M	Asthma
García-Sancho et al. (2011)	2011	United States	National Institute of Respiratory Diseases	67.8 ± 9.5	363	46	NR	U	Idiopathic PF
Emilsson et al. (2013)	2013	Iceland, Sweden and Belgium	Iceland, Sweden and Belgium	33.5 ± 7.2/34.0 ± 6.8/35.0 ± 7.3	1,560	262	NR	U	Asthma
Patria et al. (2013)	2013	Italy	Department of Pathophysiology and Transplantation, University of Milan	7.9 ± 4.5/8.1 ± 4.5	291	51	NR	M	Pneumonia
Martinez et al. (2014)	2014	United States	COPDGene Study	63.1 ± 8.6	4,483	1,307	NR	U	Asthma, pneumonia
Wang et al. (2014)	2014	China	Zhejiang Tongde Hospital	72.3 ± 5.6	876	314	NR	M	Pneumonia
Zhong et al. (2015)	2015	China	People’s Hospital of Ganzhou	NR	1,309	349	NR	M	ALI
Fan et al. (2016)	2016	China	Taiwan’s National Health Insurance Research Database	51 (39–64)	63,930	31,965	NR	M	PTB
Hsu et al. (2016)	2016	China	Taiwan’s National Health Insurance Research Database	18–100	76,369	15,412	NR	M	Lung cancer
Yang et al. (2016)	2016	China	Second Hospital of Tianjin Medical University	76.1 ± 5.8	986	350	NR	U	Pneumonia
Hsu et al. (2017)	2017	China	Taiwan’s National Health Insurance Research Database	48.3 ± 16.0	31,430	15,715	NR	M	Pneumonia
Zhang et al. (2017)	2017	China	Xianyang Central Hospital	71.2 ± 5.3	120	38	NR	M	Pneumonia
Choi et al. (2019)	2019	Republic of Korea	Korean National Health Insurance Database	≥20	1,070	427	NR	M	Lung cancer
Kim et al. (2020a)	2020	Korea	Korean Health Insurance Review and Assessment Service-National Sample Cohort	≤14	1,596	532	NR	M	Asthma
Kim et al. (2020b)	2020	Korea	Korean Health Insurance Review and Assessment Service-National Sample Cohort	≥20	349,506	116,502	NR	M	Asthma
Kuo et al. (2020)	2020	China	Taiwan’s National Health Insurance Research Database	5.8 ± 4.6/3.9 ± 3.6/3.6 ± 3.9	6,356	220	NR	M	Pneumonia
Wang et al. (2020)	2020	China	Taiwan’s National Health Insurance Research Database	55.8 ± 15.5/56.7 ± 15.1	48,154	685	NR	U	Asthma
Abdel Baseer and Sakhr (2021)	2021	Egypt	South Valley University hospital	7.17 ± 1.72/6.59 ± 1.80	174	20	NR	M	Pneumonia
Baqir et al. (2021)	2021	Minnesota	Rochester Epidemiology Project	74 (67–80)	339	113	NR	M	PF
Cantarutti et al. (2021)	2021	Italy	Italian primary care database	Children	454,113	9,020	Treated/ untreated	M	Asthma
Amarnath et al. (2022)	2022	United States	17 Northwell healthcare facilities	72.9 ± 13.1/71.7 ± 10.7	1,083	174	Mixed/treated	M	Lung cancer
Ahn et al. (2023)	2023	United States	United Kingdom Biobank	NR	332,601	71,522	NR	U	Asthma
Cotton et al. (2023)	2023	United Kingdom	NR	NR	11,259	2,668	NR	M	PF
Duncan et al. (2023)	2023	United States	Aerodigestive Center at Boston Children’s Hospital	7.9 ± 0.5	43	20	NR	U	Bronchiectasis
Jareebi et al. (2023)	2023	SAU	Integrative Epidemiology Unit, FinnGen	NR	602,604	NR	NR	U	ILD
Kim et al. (2023)	2023	Republic of Korea	Korean National Health Insurance Service National Sample Cohort	≥20	87,120	17,424	NR	U	NTMPD
Li et al. (2023b)	2023	China	Integrative Epidemiology Unit, FinnGen	NR	602,604	129,080	NR	U	Lung cancer
Reynolds et al. (2023)	2023	United Kingdom	FinnGen, International Lung Cancer Consortium	NR	367,441	78,707	NR	U	Idiopathic PF
Sarwar Zubairi et al. (2023)	2023	Pakistan	Aga Khan University Hospital (AKUH) and Jinnah Postgraduate Medical Centre	66.1 ± 10.9/64.6 ± 11.1	454	121	NR	U	Idiopathic PF
Shen et al. (2023)	2023	China	Integrative Epidemiology Unit, FinnGen	NR	602,604	129,080	NR	U	COPD
Cleven et al. (2024)	2024	United States	Population of firefighters and EMS providers at the WTC-disaster site	40 ± 9.5/53 ± 8.2	14,525	6,873	NR	M	ILD
Dong et al. (2024)	2024	China	Integrative Epidemiology Unit, FinnGen	NR	602,604	129,080	NR	U	Bronchitis, pneumonia, pulmonary embolism
Fakhraei et al. (2024)	2024	Iceland	Respiratory Health in Northern Europe	25–54	11,024	1,323	NR	M	Asthma
Liu et al. (2024)	2024	China	United Kingdom Biobank	NR	332,601	71,522	NR	U	COPD
Shan and Ge (2024)	2024	China	China-Japan Friendship Hospital	46 ± 14.5	59	33	NR	M	ILD
Wu et al. (2024)	2024	China	Integrative Epidemiology Unit, FinnGen	NR	602,604	129,080	NR	U	Idiopathic PF
Yao et al. (2024)	2024	China	Integrative Epidemiology Unit, FinnGen	NR	602,604	129,080	NR	U	Asthma

NR, not reported; U, univariate analysis; M, multivariate analysis; GERD, gastroesophageal reflux disease; OR, odds ratio; COPD, chronic obstructive pulmonary disease; PF, pulmonary fibrosis; ALI, acute lung injury; PTB, pulmonary tuberculosis; ILD, interstitial lung disease; NTMPD, non-tuberculous mycobacteria pulmonary disease.

Detailed information on the methodological quality assessment is presented in Supplementary Table 1. All the included studies were of high quality with NOS scores ≥6.

### Meta-analysis results for primary outcomes

**FIGURE 2:**
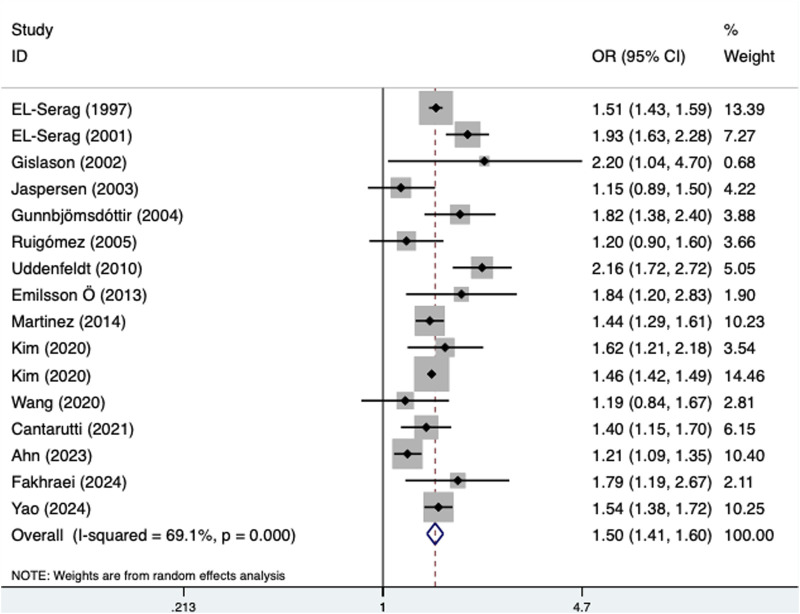
Association of the presence of gastroesophageal reflux disease with the incidence of asthma.

The pooled results demonstrated that the presence of GERD was significantly associated with an increased incidence of asthma (OR = 1.50, 95% CI: 1.41–1.60, P < 0.001; I^2^ = 69.1%, P < 0.001) (Figure 2). In addition, subgroup analyses stratified by age (adult: OR = 1.48, 95% CI: 1.41–1.55, P < 0.001; child: OR = 1.65, 95% CI: 1.33–2.04, P < 0.001) (Supplementary Table S1A) and source of OR (multivariate: OR = 1.56, 95% CI: 1.44–1.69, P < 0.001; univariate: OR = 1.39, 95% CI: 1.23–1.58, P < 0.001) (Supplementary Table S1B) revealed similar findings. However, a subgroup analysis on the basis of treatment history revealed a significant association between GERD and the incidence of asthma only among untreated patients (OR = 1.57, 95% CI: 1.19–2.08; P = 0.002) (Table 2).

**TABLE 2:** Results of meta-analysis for primary outcomes.

Items	Number of studies	OR	95% CI	P Value	I^2^ (%)	P Value for heterogeneity
Asthma	16 el-Serag and Sonnenberg. (1997), El-Serag et al. (2001), Gislason et al. (2002), Jaspersen et al. (2003), Gunnbjömsdóttir et al. (2004), Ruigómez et al. (2005), Uddenfeldt et al. (2010), Emilsson et al. (2013), Martinez et al. (2014), Kim et al. (2020a), Kim et al. (2020b), Wang et al. (2020), Cantarutti et al. (2021), Ahn et al. (2023), Fakhraei et al. (2024), Yao et al. (2024)	1.50	1.41–1.60	<0.001	69.1	<0.001
Treatment of GERD						
Treated	1 Cantarutti et al. (2021)	1.27	0.97–1.66	0.081	-	-
Untreated	1 Cantarutti et al. (2021)	1.57	1.19–2.08	0.002	-	-
Age						
Adult	9 el-Serag and Sonnenberg. (1997), Gislason et al. (2002), Jaspersen et al. (2003), Gunnbjömsdóttir et al. (2004), Emilsson et al. (2013), Martinez et al. (2014), Kim et al. (2020b), Wang et al. (2020), Fakhraei et al. (2024)	1.48	1.41–1.55	<0.001	31.3	0.168
Child	3 El-Serag et al. (2001), Kim et al. (2020a), Cantarutti et al. (2021)	1.65	1.33–2.04	<0.001	66.8	0.049
Source of OR						
Multivariate	11 el-Serag and Sonnenberg. (1997), El-Serag et al. (2001), Gislason et al. (2002), Jaspersen et al. (2003), Gunnbjömsdóttir et al. (2004), Ruigómez et al. (2005), Uddenfeldt et al. (2010), Kim et al. (2020a), Kim et al. (2020b), Cantarutti et al. (2021), Fakhraei et al. (2024)	1.56	1.44–1.69	<0.001	69.0	<0.001
Univariate	5 Emilsson et al. (2013), Martinez et al. (2014), Wang et al. (2020), Ahn et al. (2023), Yao et al. (2024)	1.39	1.23–1.58	<0.001	68.2	0.013
Pneumonia	11 el-Serag and Sonnenberg. (1997), El-Serag et al. (2001), Patria et al. (2013), Martinez et al. (2014), Wang et al. (2014), Yang et al. (2016), Hsu et al. (2017), Zhang et al. (2017), Kuo et al. (2020), Abdel Baseer and Sakhr. (2021), Dong et al. (2024)	1.53	1.35–1.74	<0.001	89.3	<0.001
Age						
Adult	6 el-Serag and Sonnenberg. (1997), Martinez et al. (2014), Wang et al. (2014), Yang et al. (2016), Hsu et al. (2017), Zhang et al. (2017)	1.45	1.25–1.68	<0.001	90.3	<0.001
Child	4 El-Serag et al. (2001), Patria et al. (2013), Kuo et al. (2020), Abdel Baseer and Sakhr. (2021)	2.04	1.45–2.88	<0.001	61.1	0.052
Source of OR						
Multivariate	8 el-Serag and Sonnenberg. (1997), El-Serag et al. (2001), Patria et al. (2013), Wang et al. (2014), Hsu et al. (2017), Zhang et al. (2017), Kuo et al. (2020), Abdel Baseer and Sakhr. (2021)	1.84	1.42–2.39	<0.001	90.6	<0.001
Univariate	3 Martinez et al. (2014), Yang et al. (2016), Dong et al. (2024)	1.31	1.13–1.51	<0.001	81.6	0.004

OR, odds ratio; CI, confidence interval; GERD, gastroesophageal reflux disease.

**FIGURE 3:**
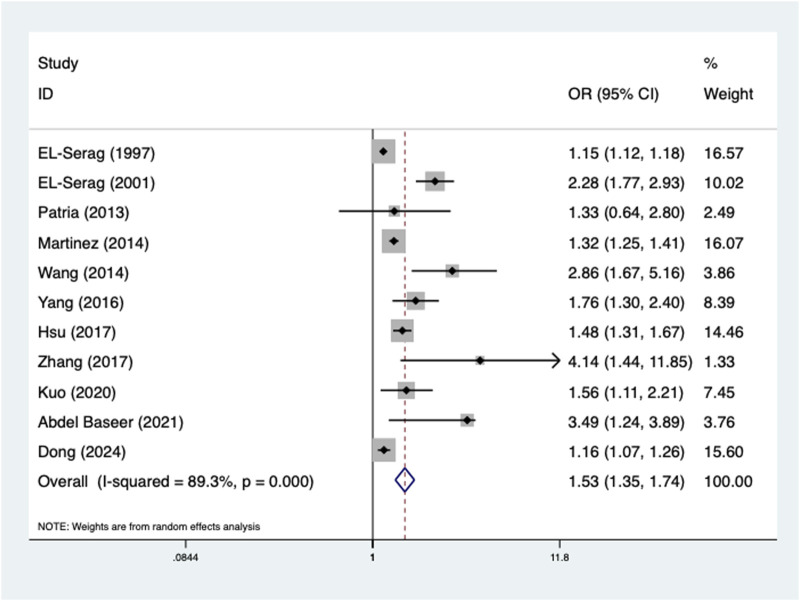
Association of the presence of gastroesophageal reflux disease with the incidence of pneumonia.

Furthermore, the presence of GERD was also related to increased incidence of pneumonia (OR = 1.53, 95% CI: 1.35–1.74, P < 0.001; I^2^ = 89.3%, P < 0.001) (Figure 3), which was confirmed by subgroup analyses according to age (adult: OR = 1.45, 95% CI: 1.25–1.68, P < 0.001; child: OR = 2.04, 95% CI: 1.45–2.88, P < 0.001) (Supplementary Table S1C) and the source of OR (multivariate: OR = 1.84, 95% CI: 1.42–2.39, P < 0.001; univariate: OR = 1.31, 95% CI: 1.13–1.51, P < 0.001) (Supplementary Table S1D).

### Meta-analysis results for secondary outcomes

According to the currently available data, GERD significantly increased the incidence of PF (OR = 1.43, 95% CI: 1.17–1.76, P = 0.001; I^2^ = 92.6%, P < 0.001), COPD (OR = 1.41, 95% CI: 1.12–1.78, P = 0.004; I^2^ = 90.6%, P < 0.001), lung cancer (OR = 1.51, 95% CI: 1.33–1.71, P < 0.001; I^2^ = 28.7%, P = 0.240), ILD (OR = 1.28, 95% CI: 1.05–1.57, P = 0.015; I^2^ = 94.4%, P < 0.001), bronchiectasis (OR = 1.63, 95% CI: 1.02–2.60, P = 0.039; I^2^ = 51.5%, P = 0.127), bronchitis (OR = 1.24, 95% CI: 1.16–1.33, P < 0.001; I^2^ = 64.4%, P = 0.094), ALI (OR = 2.07, 95% CI: 1.37–2.89, P < 0.001), pulmonary embolism (OR = 1.33, 95% CI: 1.12–1.58, P = 0.013), PTB (OR = 1.63, 95% CI: 1.10–2.40, P = 0.015) and NTMPD (OR = 3.36, 95% CI: 2.10–5.37, P < 0.001) (Table 3).

**TABLE 3:** Results of meta-analysis for secondary outcomes.

Items	Number of studies	OR	95% CI	P Value	I^2^ (%)	P Value for heterogeneity
PF	8 el-Serag and Sonnenberg. (1997), Gribbin et al. (2009), García-Sancho et al. (2011), Baqir et al. (2021), Cotton et al. (2023), Reynolds et al. (2023), Sarwar Zubairi et al. (2023), Wu et al. (2024)	1.43	1.17–1.76	0.001	92.6	<0.001
Source of OR						
Multivariate	3 el-Serag and Sonnenberg. (1997), Baqir et al. (2021), Cotton et al. (2023)	1.38	1.27–1.50	<0.001	0.0	0.428
Univariate	5 Gribbin et al. (2009), García-Sancho et al. (2011), Reynolds et al. (2023), Sarwar Zubairi et al. (2023), Wu et al. (2024)	1.42	1.03–1.96	0.031	88.0	<0.001
COPD	4 el-Serag and Sonnenberg. (1997), Garcia Rodriguez et al. (2008), Shen et al. (2023), Liu et al. (2024)	1.41	1.12–1.78	0.004	90.6	<0.001
Source of OR						
Multivariate	2 el-Serag and Sonnenberg. (1997), Garcia Rodriguez et al. (2008)	1.22	1.17–1.27	<0.001	0.0	0.744
Univariate	2 Shen et al. (2023), Liu et al. (2024)	1.66	1.15–2.40	0.007	89.1	0.002
Lung cancer	4 (Hsu et al. (2016), Choi et al. (2019), Amarnath et al. (2022), Li et al. (2023b)	1.51	1.33–1.71	<0.001	28.7	0.240
Treatment of GERD						
Treated	1 Amarnath et al. (2022)	1.01	0.48–2.12	0.97	-	-
Source of OR						
Multivariate	3 Hsu et al. (2016), Choi et al. (2019), Amarnath et al. (2022)	1.70	1.40–2.05	<0.001	0.0	0.471
Univariate	1 Li et al. (2023b)	1.37	1.16–1.62	<0.001	-	-
ILD	3 Jareebi et al. (2023), Cleven et al. (2024), Shan and Ge. (2024)	1.28	1.05–1.57	0.015	94.4	<0.001
Source of OR						
Multivariate	2 Cleven et al. (2024), Shan and Ge. (2024)	4.96	2.83–8.69	<0.001	0.0	0.913
Univariate	1 Jareebi et al. (2023)	1.09	0.94–1.25	0.250	95.6	<0.001
Bronchiectasis	3 el-Serag and Sonnenberg. (1997), El-Serag et al. (2001), Duncan et al. (2023)	1.63	1.02–2.60	0.039	51.5	0.127
Age						
Adult	1 el-Serag and Sonnenberg. (1997)	1.26	1.08–1.46	0.002	-	-
Child	2 El-Serag et al. (2001), Duncan et al. (2023)	2.30	1.31–4.05	0.004	0.0	0.960
Bronchitis	2 el-Serag and Sonnenberg. (1997), Dong et al. (2024)	1.24	1.16–1.33	<0.001	64.4	0.094
ALI	1 Zhong et al. (2015)	2.07	1.37–2.89	<0.001	-	-
Pulmonary embolism	1 Dong et al. (2024)	1.33	1.12–1.58	0.013	-	-
PTB	1 Fan et al. (2016)	1.63	1.10–2.40	0.015	-	-
NTMPD	1 Kim et al. (2023)	3.36	2.10–5.37	<0.001	-	-

OR, odds ratio; CI, confidence interval; GERD, gastroesophageal reflux disease; COPD, chronic obstructive pulmonary disease; PF, pulmonary fibrosis; ALI, acute lung injury; PTB, pulmonary tuberculosis; ILD, interstitial lung disease; NTMPD, non-tuberculous mycobacteria pulmonary disease.

Subgroup analyses stratified by the source of OR for PF (multivariate: OR = 1.38, 95% CI: 1.27–1.50, P < 0.001; univariate: OR = 1.42, 95% CI: 1.03–1.96, P = 0.031) (Supplementary Figure S2A), COPD (multivariate: OR = 1.22, 95% CI: 1.17–1.27, P < 0.001; univariate: OR = 1.66, 95% CI: 1.15–2.40, P = 0.007) (Supplementary Figure S2B), lung cancer (multivariate: OR = 1.70, 95% CI: 1.40–2.05, P < 0.001; univariate: OR = 1.37, 95% CI: 1.16–1.62, P < 0.001) (Supplementary Figure S2C) and ILD (multivariate: OR = 4.96, 95% CI: 2.83–8.69, P < 0.001) (Supplementary Figure S2D) and stratified by age for bronchiectasis (adult: OR = 1.26, 95% CI: 1.08–1.46, P = 0.002; child: OR = 2.30, 95% CI: 1.31–4.05, P = 0.004) (Supplementary Figure S2E) further clarified the associations between the occurrence of GERD and increased incidence of the above pulmonary diseases.

### Sensitivity analysis and publication bias

**FIGURE 4:**
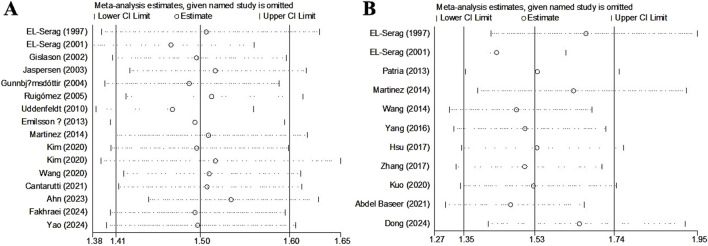
Sensitivity analysis about the association of gastroesophageal reflux disease with the incidence of asthma **(A)** and pneumonia **(B)**.

Sensitivity analyses for asthma (Figure 4A) and pneumonia (Figure 4B) indicated that the results were stable and reliable and that none of the included studies significantly affected the pooled results.

**FIGURE 5:**
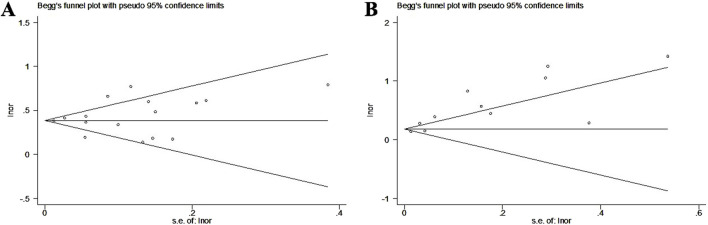
Begg’s funnel plots about the association of gastroesophageal reflux disease with the incidence of asthma **(A)** and pneumonia **(B)**.

According to Begg’s funnel plots and Egger’s test for asthma (Figure 5A, P = 0.462) and pneumonia (Figure 5B, P = 0.002), there was significant publication bias for the association between GERD and the incidence of pneumonia. Therefore, the trim-and-fill method was applied, and two potentially unpublished studies were identified (Supplementary Figure S3); however, these two studies did not affect the overall conclusion (fixed OR = 1.20, 95% CI: 1.17–1.22, P < 0.001; random OR = 1.46, 95% CI: 1.29–1.66, P < 0.001).

## Discussion

Our meta-analysis is the first to comprehensively and systematically explore the impact of GERD on the incidence of pulmonary diseases. On the basis of our pooled results, it is believed that GERD are significantly associated with increased incidence of pulmonary diseases such as asthma, pneumonia, PF and lung cancer. However, standardized treatment might reduce this incidence of pulmonary diseases. The above findings highlight the importance of screening and management for pulmonary diseases and of regular therapy in patients with GERD.

The development of pulmonary diseases related to GERD is associated with various mechanisms. During GERD, stomach acid and gastric contents can reflux into the esophagus and eventually be aspirated into the airways. These small amounts of stomach acid and other gastric contents can irritate the airway mucosa, triggering an inflammatory response that leads to airway hyperreactivity and bronchoconstriction, which induces asthma and COPD (Nemzek and Kim, 2009; Houghton et al., 2016). Chronic microaspiration may also increase the risk of infections (Thomson et al., 2007). Gastric acid reflux stimulates nerve endings in the esophagus and upper airways, activating vagal nerve reflexes that cause airway constriction and inflammation. This reflexive response can lead to asthma symptoms and increase the risk of acute COPD exacerbation (Harding, 2001; Patterson and Harding, 1999). Additionally, the local inflammatory response triggered by stomach acid releases inflammatory mediators such as cytokines and histamine, which can circulate through the bloodstream and affect the lungs, exacerbating airway inflammation, worsening asthma and COPD, and increasing the risk of pulmonary infections (Azer et al., 2024). Long-term acid reflux can cause damage to the mucosa of both the upper and lower airways, weakening local defense mechanisms and increasing the susceptibility of the airways to pathogen infections. This mucosal damage can trigger or worsen pneumonia and may promote the development of pulmonary fibrosis (Houghton et al., 2016). The inflammation caused by GERD may extend downward into the airways, leading to chronic inflammation. Persistent chronic inflammation can not only cause pneumonia but also contribute to the development of lung cancer (Demb et al., 2019).

As mentioned above, the microaspiration of stomach acid and gastric contents into the airways is the core cause of the subsequent development of pulmonary diseases in patients with GERD. Therefore, standardized acid suppression therapy is crucial for reducing the risk of pulmonary diseases in GERD patients. This is consistent with our findings, as patients who complied with regular treatment were not at increased incidence of pulmonary diseases. Furthermore, we believe that improving lifestyle habits, such as dietary adjustments, maintaining proper postmeal positioning, weight management, and avoiding smoking and excessive alcohol consumption, could also help to control the symptoms of GERD, which would contribute to reducing the incidence of subsequent pulmonary diseases (Katz et al., 2022; Katzka and Kahrilas, 2020).

In our meta-analysis, the inclusion criteria were strictly defined and required that GERD be diagnosed prior to pulmonary diseases or similar descriptions in the articles were required to better evaluate the impact of the presence of GERD on the incidence of pulmonary diseases. Some pulmonary diseases can also affect the incidence and exacerbation of GERD. Patients with asthma, COPD and PF are at increased incidence of GERD (Ruigómez et al., 2005; Liang and Feng, 2012; Dziekiewicz et al., 2015; Ruaro et al., 2022). The key causes include the following. Asthma and COPD can affect patients’ breathing, especially during an asthma attack or an acute exacerbation of COPD. Forceful breathing and frequent coughing increase the pressure in the chest and abdomen, which pushes stomach contents back into the esophagus, triggering GERD (Ayazi et al., 2011; Solidoro et al., 2017). Asthmatic patients and COPD patients often experience frequent coughing and an increase in the volume of respiratory secretions. Repeated coughing exerts pressure on the lower esophageal sphincter (LES), which can cause it to relax (Azer et al., 2024). When the LES relaxes, its ability to prevent stomach acid from refluxing weakens, leading to an increased incidence of acid reflux (Azer et al., 2024). Patients with severe pulmonary diseases may have reduced physical activity and are more frequently in a reclining or semireclining position, which increases the risk of acid reflux, especially after eating or during sleep. Some studies suggest that lung diseases can trigger esophageal motility disorders through vagal nerve reflexes. This reflex response can lead to decreased LES function, thereby increasing the risk of acid reflux (Zhang et al., 2021). Therefore, studies in which pulmonary diseases were diagnosed prior to GERD and cross-sectional studies were excluded from our meta-analysis.

Our results suggested that standardized treatment could reduce the impact of GERD on the incidence of pulmonary diseases. However, we believe this pattern may be more applicable to GERD patients who have been certainly diagnosed through gastroscopy, PPI test, or 24-h esophageal pH monitoring rather than the symptoms, as well as patients with risk factors such as the elderly age and obesity for pulmonary diseases. Besides, several studies investigating the associations of the presence of GERD with exacerbdations of asthma, pneumonia and COPD among GERD patients have been conducted, and GERD has been reported to be a risk factor for poor disease control (Ferrera et al., 2024; Gaillet et al., 2015; Hozawa et al., 2022; Baumeler et al., 2016; Bigatao et al., 2018). However, few studies have explored the relationship between the treatment of GERD and pulmonary disease control. There are many GERD patients with pulmonary diseases in the clinic. Therefore, further clarification of the impact of standardized treatment for GERD on the exacerbation of lung diseases is crucial, as this information will help improve the clinical management of these patients.

In several studies, specific populations were involved, such as firefighters and EMS providers at the WTC disaster site (Cleven et al., 2024) and children and young people with cerebral palsy (Kuo et al., 2020), which may affect the generalizability of our conclusions. However, the majority of enrolled patients were from the general population and the inclusion of these studies was intended to capture a broad spectrum of GERD-related impacts across different contexts, thereby enriching our understanding of the association between GERD and development of pulmonary diseases in diverse populations. Substantial heterogeneity was observed in several analyses, which may be related to various factors such as diagnostic criteria, study populations, or designs. However, due to the lack of original data, this could not be confirmed. Therefore, more detailed analyses are needed in future related studies to further determine our findings. Furthermore, significant publication bias about the association between GERD and incidence of pneumonia was detected, which may be related to a bias toward positive results, sample selection bias (i.e., some studies focusing on populations more prone to pneumonia), and the impact of small-sample studies. However, the analysis of trim-and-fill method indicated that the potentially unpublished studies did not affect the results and our conclusion was stable and reliable.

There are several limitations in our meta-analysis. First, most of the included studies were retrospective, and the sample sizes of ten studies were fewer than one thousand, which might cause some bias. Second, in the eighteen included studies, only univariate analyses were performed, and we were unable to conduct subgroup analyses on the basis of other important parameters, such as gastrointestinal comorbidities, the degree of GERD and lifestyle habits, including smoking and alcohol consumption, due to the lack of original data. Therefore, the impacts of these confounding factors on the association of GERD with the incidence of pulmonary diseases need to be investigated. Third, few studies have explored the relationships between the presence of GERD and the incidences of COPD (n = 4), lung cancer (n = 4), ILD (n = 3), bronchiectasis (n = 3), ALI (n = 1), pulmonary embolism (n = 1), PTB (n = 1) and NTMPD (n = 1) and only three studies reported on GERD treatment, which should be evaluated by future studies. Fourth, our results indicate that standardized treatment may eliminate the impact of GERD on the incidence of pulmonary diseases. However, the drugs and treatment periods used were not further reviewed or analyzed. Fifth, not all patients were diagnosed by gastroscopy, PPI test or 24-h esophageal pH monitoring, rather, diagnoses were made based on symptoms, which may lead to a certain degree of bias. Sixth, in a few studies, specific populations were included, such as firefighters and EMS providers at the WTC disaster site (Cleven et al., 2024) and children and young people with cerebral palsy (Kuo et al., 2020), which may affect the generalizability of our conclusions. However, the majority of enrolled patients were from the general population. Seventh, some included studies lacked explicit information on the temporal sequence of GERD diagnosis and pulmonary disease onset. Although we inferred directionality from study design and language, the absence of concrete timing data may introduce uncertainty in causal interpretation. Eighth, furthermore, although many included studies reported GERD diagnosis prior to the diagnosis of pulmonary diseases, this does not guarantee that GERD preceded the actual onset of pulmonary pathology, given the possibility of diagnostic delays, especially in chronic or asymptomatic lung conditions.

## Conclusion

Overall, GERD is associated with increased incidence of various pulmonary diseases, but regular treatment may eliminate this effect. These findings indicate the importance of screening and management for pulmonary diseases and standardized therapy in patients with GERD. However, owing to the limitations of our meta-analysis, further prospective high-quality studies are needed to verify the findings, and more detailed investigations should be performed.

## Data Availability

The original contributions presented in the study are included in the article/Supplementary Material, further inquiries can be directed to the corresponding authors.
