# Correction: Association of gastroesophageal reflux disease with the incidence of pulmonary disease

**DOI:** 10.3389/fcell.2025.1719856

**Published:** 2025-10-22

**Authors:** Xin Wang, Yan Wang, Yijie Bu, Yu Liu, Sheng Gong, Guowei Che

**Affiliations:** ^1^ Department of Respiratory and Critical Care Medicine, State Key Laboratory of Respiratory Health and Multimorbidity, Key Laboratory of Plateau Medicine of Sichuan Province, Center for High Altitude Medicine, West China Hospital, Sichuan University, Chengdu, China; ^2^ Department of Thoracic Surgery/Lung Cancer Center, West China Hospital, Sichuan University, Chengdu, China; ^3^ Department of Thoracic Surgery, The Public Health Clinical Center of Chengdu, Chengdu, China

**Keywords:** gastroesophageal reflux disease, pulmonary disease, meta-analysis, asthma, pneumonia

**Affiliation 1** “Department of Respiratory and Critical Care Medicine, State Key Laboratory of Respiratory Health and Multimorbidity, Key Laboratory of Plateau Medicine of Sichuan Province, Center for High Altitude Medicine, West China Hospital, Sichuan University, Chengdu, China” was erroneously given as 1 “Department of Respiratory and Critical Care Medicine, State Key Laboratory of Respiratory Health and Multimorbidity, Key Laboratory of Plateau Medicine of Sichuan Province, West China Hospital, Sichuan University, Chengdu, China”.

An incorrect number was provided for the funder “1-3-5 Project of Center for High Altitude Medicine, West China Hospital, Sichuan University”. The correct number is GYYX24008. The funder “1-3-5 Project for Disciplines of Excellence, West China Hospital, Sichuan University, (ID: ZYAI24001 to GC)” was erroneously omitted.

The original article has been updated.

